# Immunization with Heterologous Flaviviruses Protective Against Fatal West Nile Encephalitis

**DOI:** 10.3201/eid0803.010238

**Published:** 2002-03

**Authors:** Robert B. Tesh, Amelia P.A. Travassos da Rosa, Hilda Guzman, Tais P. Araujo, Shu-Yuan Xiao

**Affiliations:** University of Texas Medical Branch, Galveston, Texas, USA

**Keywords:** West Nile virus, viral encephalitis, arboviruses, Flavivirus, experimental infection, immune response, immunization, Japanese encephalitis virus, vaccines

## Abstract

Prior immunization of hamsters with three heterologous flaviviruses (*Japanese encephalitis virus* [JEV] SA14-2-8 vaccine, wild-type *St. Louis encephalitis virus* [SLEV], and Y*ellow fever virus* [YFV] 17D vaccine) reduces the severity of subsequent *West Nile virus* (WNV) infection. Groups of adult hamsters were immunized with each of the heterologous flaviviruses; approximately 30 days later, the animals were injected intraperitoneally with a virulent New York strain of WNV. Subsequent levels of viremia, antibody response, and deaths were compared with those in nonimmune (control) hamsters. Immunity to JEV and SLEV was protective against clinical encephalitis and death after challenge with WNV. The antibody response in the sequentially infected hamsters also illustrates the difficulty in making a serologic diagnosis of WNV infection in animals (or humans) with preexisting *Flavivirus* immunity.

*West Nile virus* (WNV) was detected for the first time in North America in summer of 1999, during an outbreak involving humans, equines, and birds in the New York City metropolitan area (*1*). Persistence of the virus and its spread to other states on the eastern seaboard during 2000 and 2001 suggest that WNV is now endemic in the United States and that its geographic range probably will continue to expand until it extends over much of the continent (*2*). Although many WNV infections in humans are asymptomatic or unrecognized, some patients have an acute dengue-like illness, and a small percentage have encephalitis or meningoencephalitis (*1*–*5*). The latter complication is most common among the elderly, with recent reported case-fatality rates from 4% to 11% (*3*–*9*). No specific treatment is available for WNV encephalitis, and no licensed vaccine is available for its prevention.

WNV is a positive-stranded RNA virus; based on its antigenic and genetic characteristics, it is included in the *Japanese encephalitis virus* (JEV) serocomplex of the genus *Flavivirus*, family *Flaviviridae* (*10*). The JEV serocomplex includes four antigenically related viruses that are important causes of encephalitis in humans: JEV, WNV, *St. Louis encephalitis virus* (SLEV), and *Murray Valley encephalitis virus* (MVEV). In addition to their antigenic and genetic relatedness, these four viruses have many epidemiologic similarities (*3*,*11*).

Because of the close antigenic relationships among many viruses in this genus, *Flavivirus* infections are difficult to differentiate by most serologic techniques, especially in persons or animals having a second or sequential *Flavivirus* infection (*12*–*14*). Considerable attention has been focused on the immune response in primary and secondary *Flavivirus* infection and the role of immunopathogenesis in the etiology of severe *Flavivirus* disease (*11*,*15*,*16*). In the case of dengue, enhancement of virus replication by heterologous flavivirus antibodies and T-cell activation are thought to occur in some patients during a second or sequential dengue infection, resulting in hemorrhagic fever or shock (*15*,*16*). In contrast, animal data indicate that prior infection with a heterologous *Flavivirus* reduces the severity of subsequent challenge with WNV. Results of experimental studies with rodents, monkeys, and pigs (*17*–*21*) suggest that heterologous *Flavivirus* antibodies protect against or modify subsequent infection with WNV. This phenomenon could be important in vaccine development against WNV infection and in determining the ultimate geographic distribution and public health importance of WNV if it is introduced into areas of Central and South America where other flaviviruses, such as *Dengue virus* (DENV), Y*ellow fever virus* (YFV), SLEV, and *Ilhéus virus* (ILHV), are endemic.

To determine more precisely the degree of cross-protection among members of the JEV serocomplex and the possibility that this phenomenon could be used to protect against severe WNV infection, a series of experiments was carried out with three heterologous flaviruses and a recently described model (*22*) of WNV encephalitis. We report the results of these studies, which indicate that prior immunization of hamsters with a JEV vaccine strain and a wild-type SLEV--and to a lesser extent the 17-D YFV vaccine--modify subsequent WNV infection and protect the animals from fatal encephalitis.

## Materials and Methods

Four flaviviruses were used in this study: WNV strain 385-99, isolated from a dead snowy owl at the Bronx Zoo during the 1999 epizootic in New York City (*23*); live attenuated SA14-2-8 vaccine strain of JEV (*24*,*25*); 17-D live attenuated vaccine strain of YFV (*26*); and SLEV strain Be Ar 23379 (*27*), originally isolated from mosquitoes (*Sabethes belisarioi*) in Para, Brazil, in 1961.

The hamsters used in our studies were adult (70 g to 100 g) female Syrian golden hamsters (*Mesocricetus auratus*) (Harlan Sprague Dawley, Indianapolis, IN). Animals were cared for in accordance with the guidelines of the Committee on Care and Use of Laboratory Animals (Institute of Laboratory Animal Resources, National Research Council) under an animal use protocol approved by the University of Texas Medical Branch. All work with infected animals was carried out in biosafety level-3 facilities.

All virus titrations were done in cultures of the C6/36 clone of *Aedes albopictus* cells (*28*), with the presence or absence of viral antigen by immunofluorescence as the endpoint, as described (*22*,*29*,*30*). To determine the quantity of infectious virus in blood samples taken daily after WNV infection, each hamster blood specimen was titrated in 24-well tissue culture plates seeded with C6/36 cells. Serial 10-fold dilutions from 10^-1^ to 10^-7^ were made of each sample in phosphate-buffered saline, pH 7.4 (PBS), containing 10% fetal bovine serum; 0.1 mL of each dilution was added to four wells of a tissue culture plate. Following absorption at 28EC for 2 hours, 1.5 mL of maintenance medium (*29*) was added to each well, and the plates were incubated at 28EC in a 5% CO_2_ atmosphere for 6 days. On day 6, 20 mL of a cell suspension from each well was added to a single spot on 12-spot glass microscope slides (Cell-Line Associates, Inc., Newfield, NJ). After drying at room temperature, the slides were immersed in cold acetone for 10 minutes; the cells were subsequently examined for the presence of WNV antigen by indirect fluorescent antibody test by using a WNV-specific mouse immune ascitic fluid (see below) and a commercially prepared fluorescein-conjugated, goat antimouse immunoglobulin (Sigma, St. Louis, MO). WNV titers were calculated as the tissue culture infectious dose_50_ (TCID_50_) per mL of specimen by the method of Reed and Muench (*31*).

### Experimental Infection of Animals

Hamsters were infected by the intraperitoneal (IP) or subcutaneous (SC) routes, depending on the virulence of the infecting virus for the animals. WNV and YFV were injected IP; JEV and SLEV were administered SC. Infecting doses of the viruses were as follows: WNV 10^4.0^ TCID_50_, YFV 10^6.0^ TCID_50_, JEV 10^6.5^ TCID_50,_ and SLEV 10^6.0^ TCID_50_.

### Immune Reagents

A mouse immune ascitic fluid to WNV was prepared in adult mice. The immunogen was a crude homogenate of brain (10% W/V in PBS) from newborn mice injected intracerebrally (IC) with the B956 prototype strain of WNV (*32*). The adult immunization schedule consisted of four IP injections of the immunogen mixed with Freund’s adjuvant, given at weekly intervals. Sarcoma 180 cells were given after the final injection to induce ascites formation.

### Antibody Determinations

Serum antibodies to WNV and the other three flaviviruses were measured by hemagglutination-inhibition (HI) test and to WNV by immunoglobulin (Ig) M antibody capture enzyme immunoassay (MAC-ELISA) (*33*). Antigens for both serologic tests were prepared from brains of newborn mice injected IC with each of the flaviviruses; the infected brains were treated by the sucrose-acetone extraction method (*33*). Hamster sera were tested by HI at serial twofold dilutions from 1:20 to 1:5120 at pH 6.6 (WNV, JEV, and SLEV) or 6.4 (YFV) with 4 units of antigen and a 1:200 dilution of goose erythrocytes, following established protocols (*33*).

For the MAC-ELISA, microtiter plates were coated with a commercial goat anti-rat IgM (capture) antibody (Kirkegaard & Perry Laboratories, Inc., Gaithersburg, MD), diluted 1:500 in carbonate buffer, pH 9.6. All hamster sera were screened at a 1:40 dilution. The WNV antigen was also used at a 1:40 dilution. The secondary (detecter) antibody was a mouse, anti-*Flavivirus*, peroxidase-conjugated monoclonal antibody (6B6C-1) at a dilution of 1:6000. Results were read with a SPECTRA shell reader (SLT Labininstruments, Salzburg, Austria). Specimens wells were recorded as positive when the absorbance values at optical density_405_ nm of the specimen wells exceeded 0.20 after subtraction of average background absorbance of control wells (*33*).

## Results

### Infection of Nonimmune Hamsters with WNV

Several groups of *Flavivirus*-naive (control) hamsters were inoculated IP with 10^4^ TCID_50_ of WNV to determine the subsequent level and duration of viremia, immune response, and death rate. Table 1 and the Figure show the results of an experiment with a group of 10 hamsters that were bled daily for 6 consecutive days after infection with WNV. Moderate levels of virus were detected in the animals’ blood within 24 hours and persisted for 5 or 6 days. The highest blood virus titers were detected on days 2 and 3 after infection (means 10^5.2^ and 10^5.1^, respectively). HI antibodies were detected in all the animals by day 5, and the titers had increased substantially by day 6. In general, WNV-specific IgM, as detected by MAC-ELISA, appeared at approximately the same time as the HI antibodies (data not shown).

**Table 1:** Pattern of viremia and hemagglutination inhibition (HI) antibody response in 10 adult *Flavivirus*-naïve (control) hamsters, following intraperitoneal inoculation of 10^4^ TCID_50_ of *West Nile virus* (WNV)

Animal No.	Day postinoculation					
	D-1	D-2	D-3	D-4	D-5	D-6
8001	4.3^a^(0)	5.0 (0)	5.0 (0)	3.3 (0)	1.0 (1:80)	1.0 (1:320)
8002	4.7 (0)	5.5 (0)	5.2 (0)	3.5 (0)	2.5 (1:40)	0 (1:320)
8003	5.3 (0)	5.3 (0)	5.0 (0)	3.5 (0)	2.5 (1:40)	0 (1:320)
8004	2.0 (0)	5.0 (0)	5.0 (0)	4.3 (0)	2.5 (1:40)	1.0 (1:160)
8005	4.0 (0)	5.0 (0)	5.5 (0)	3.7 (0)	1.7 (1:80)	1.0 (1:320)
8006	4.6 (0)	5.2 (0)	5.7 (0)	4.3 (0)	2.7 (1:80)	0 (1:320)
8007	4.3 (0)	5.7 (0)	4.6 (0)	4.0 (0)	2.0 (1:80)	1.0 (1:320)
8008	4.2 (0)	5.8 (0)	4.8 (0)	1.8 (0)	2.0 (1:80)	0 (1:320)
8009	5.2 (0)	5.2 (0)	5.0 (0)	3.2 (0)	2.8 (1:80)	0 (1:320)
8010	4.7 (0)	4.7 (0)	5.5 (0)	3.5 (0)	1.8 (1:80)	0.7 (1:320)
Mean	4.3	5.2	5.1	3.5	2.1	0.5
SD	0.92	0.34	0.34	0.71	0.54	0.50

^a^WNV titer expressed as log_10_ TCID_50_/mL of blood. 0 <0.7. (HI antibody titer; 0 <1:20)

Table 2 shows the results of a second experiment in which 13 hamsters were infected with WNV. All the animals were bled 6 days after injection, and a subset was bled again at 31, 60, and 90 days. Six days after infection, all the animals had specific HI antibodies to WNV antigen and were negative to the other three flaviviral antigens tested (YFV, SLEV, and JEV). At this time, the animals also had a strongly positive IgM antibody response by MAC-ELISA. Thirty-one days after infection, the HI antibody response had become broadly cross-reactive with the four *Flavivirus* antigens, although the highest titer was still to WNV, and the IgM antibody had begun to decrease. A similar HI antibody pattern was observed at 60 and 90 days after infection, although by 90 days the HI titers were decreasing. Six of the nine WNV-infected hamsters gave a negative reaction in the WNV MAC-ELISA when tested 60 and 90 days after infection.

**Table 2:** Serologic response of adult hamsters to *West Nile virus* (WNV), *Yellow fever virus* (YFV), *St. Louis encephalitis virus* (SLEV), and *Japanese encephalitis virus* (JEV) antigens, at various intervals after intraperitoneal inoculation of 10^4.0^ TCID_50_ of WNV

		HI antibody titer									
Animal no.		WNV	YFV				SLEV		JEV		WN MAC-ELISA
Day 6											
8251(D^a^)	1:40				0^b^	0		0		0.633^c^	
8252	1:80				0	0		0		1.013	
8253(D)	1:160				0	0		0		0.878	
8254	1:160				0	0		0		1.090	
8255	1:80				0	0		0		0.848	
8256	1:80				0	0		0		0.840	
8257(D)	1:160				0	0		0		1.291	
8258	1:80				0	0		0		0.869	
8259(D)	1:160				0	0		0		0.939	
8260	1:80				0	0		0		0.992	
8262	1:80				0	0		0		0.748	
8263	1:80				0	0		0		0.797	
8264(D)	1:80				0	0		0		0.827	
Day 31											
8252	1:1,280			1:320		1:320		1:320		0.401	
8254	1:1,280			1:320		1:320		1:320		0.427	
8255	1:640			1:160		1:320		1:160		0.488	
8256	1:640			1:160		1:160		1:160		0.582	
8258	1:640			1:160		1:160		1:160		0.376	
8260	1:1,280			1:320		1:320		1:320		0.420	
8262	1:1,280			1:320		1:320		1:320		0.246	
8263	1:640			1:160		1:160		1:160		0.516	
Day 60											
8252	1:2,560			1:640		1:640		1:640		0.269	
8255	1:2,560			1:640		1:640		1:640		0.216	
8256	1:640			1:160		1:160		1:160		0.162	
8258	1:1,280			1:320		1:320		1:320		0.161	
8260	1:320			1:80		1:80		1:40		0.179	
8262	1:640			1:160		1:160		1:160		0.181	
Day 90											
8254	1:320			1:80		1:80		1:80		0.167	
8260	1:640			1:80		1:80		1:80		0.217	
8262	1:640			1:80		1:160		1:160		0.184	

^a^(D): Hamster died of encephalitis 7 to 14 days after infection. HI = hemagglutination inhibition. ^b^0 <1:20. ^c^Optical density value (>0.200 is positive).

Five of the 13 hamsters infected in this second experiment died of WNV encephalitis 7 to 14 days after infection (Table 2). Overall, 14 (47%) of 30 adult hamsters injected IP with 10^4^ TCID_50_ of WNV died of encephalitis (Table 3). The pathologic reaction of the WNV hamster model has been described (*22*).

**Table 3:** Infection and mortality rates, following intraperitoneal inoculation of 10^4^ TCID_50_ of *West Nile virus* (WNV), in nonimmune (control) hamsters, and in hamsters previously immunized with Japanese encephalitis (JE) SA14-2-8 vaccine, *St. Louis encephalitis virus* (SLEV) strain BeAr 23379, or yellow fever (YF) 17D vaccine

Immune group	No. infected with WNV	No. infected (%)^a^	No. died (%)
Nonimmune	30	30 (100)	14 (47)
JEV SA14-2-8	30	30 (100)	0 (0)
SLEV BeAr 23379	32	32 (100)	0 (0)
YFV 17D	30	30 (100)	4 (13)

^a^ Total number of animals infected or dead after being infected with WNV.

### Infection of JEV-Immune Hamsters with WNV

The Figure and Table 4 show the results from another experiment in which 30 adult hamsters were given a single SC injection of approximately 10^6.4^ TCID_50_ of the live attenuated JEV SA14-2-8 vaccine strain. Thirty-eight days later, the animals were injected (challenged) IP with 10^4^ TCID_50_ of WNV; 10 of the hamsters in this group were bled daily for 6 consecutive days. These blood samples were subsequently titrated to determine the level of WNV viremia. The resulting viremia in the JEV-immune animals was markedly lower than in the naïve hamsters (Figure). Furthermore, the JEV-immune hamsters responded to challenge with WNV by developing a secondary (sequential) type of *Flavivirus* antibody response. Table 4 shows the HI antibody titers to JEV and WNV antigens in sera of 10 of the SA14-2-8 vaccinated hamsters, 30 days after their JEV immunization. At this time the HI antibody titers to JEV and WNV antigens were characteristic of a primary *Flavivirus* infection (*13*,*14*). On day 38, the animals were challenged with WNV; 6 days later, their sera were tested for HI and WNV-specific IgM antibodies. The boost in HI antibody titers that was observed 6 days after challenge with WNV was typical of a secondary antibody response to *Flavivirus* infection (*13*,*14*). In contrast, IgM antibody response to the second *Flavivirus* (WNV) infection was minimal (Table 4).

**Table 4:** Serologic response of hamsters following immunization with the SA14-2-8 vaccine strain of *Japanese encephalitis virus* (JEV) and subsequent challenge with *West Nile virus* (WNV*)*

Hamster no.	HI antibody 30 days after JEV immunization		HI antibody 6 days after WNV challenge		WN MAC-ELISA 6 days after WNV challenge
	JEV	WNV	JEV	WNV	
8236	1:80	1:80	1:640	1:640	0.166^a^
8237	1:40	1:80	1:320	1:320	0.205
8238	1:80	1:80	1:640	1:640	0.239
8239	1:80	1:80	1:640	1:640	0.173
8240	1:80	1:80	1:640	1:640	0.245
8241	1:80	1:80	1:320	1:640	0.271
8242	1:80	1:80	1:1,280	1:1,280	0.209
8243	1:40	1:80	1:160	1:160	0.205
8244	1:40	1:80	1:320	1:320	0.229
8245	1:80	1:80	1:160	1:160	NT

^a^Optical density value (³0.200 is positive). HI = hemagglutination inhibition. NT = not tested.

**Figure:**
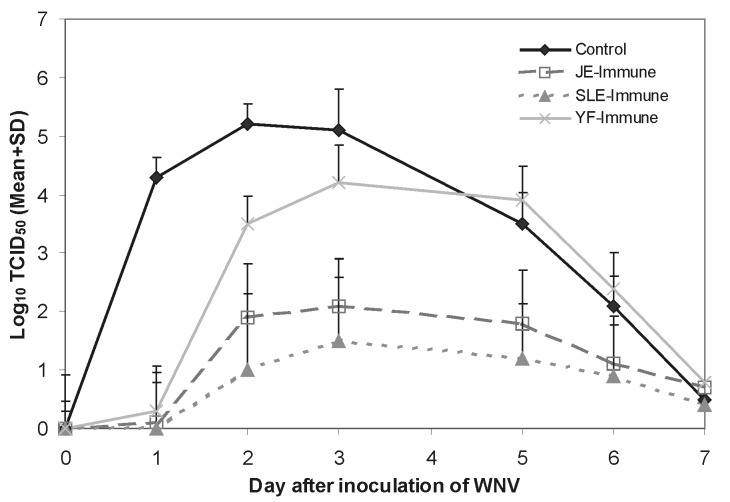
Summary of mean (±SD) *West Nile virus* (WNV) titers in daily blood samples from four groups of 10 hamsters each (control, *Japanese encephalitis virus* [JEV]-immune, *St. Louis encephalitis virus* [SLEV]-immune, and Y*ellow fever virus* [YFV]-immune) after intraperitoneal inoculation of 10^4^ tissue culture infective dose (TCID)_50_ of WNV. Mean virus titers are expressed as log_10_ TCID_50_/mL of blood.

All the JEV-immune hamsters (n = 30) survived challenge with WNV (Table 3). Their infection with WNV was confirmed by the presence of low-level viremia (Figure) and the secondary *Flavivirus* antibody response following challenge (Table 4). None of these hamsters appeared clinically ill after infection with WNV, in contrast to the naïve animals. Many of the nonimmune hamsters had clinical signs of acute central nervous system injury (somnolence, muscle weakness, paralysis, tremors, and loss of balance) beginning around day 6 after infection, and approximately half died (*22*). Thus, prior immunization with JEV vaccine reduced the severity of subsequent WNV infection and prevented death.

### Infection of SLEV-Immune Hamsters with WNV

The Figure and Table 5 summarize the results of another experiment in which 32 adult hamsters were given a single SC injection of approximately 10^6^ TCID_50_ of SLEV strain BeAr 23379. This wild-type SLEV strain was selected for immunization, since it is not lethal to hamsters. Thirty-two days after injection with SLEV, the animals were inoculated IP with 10^4^ TCID_50_ of WNV. After this WNV challenge, the hamsters were bled daily for 6 consecutive days, as before. Antibody determinations were also done on blood samples taken 6 days after challenge with WNV.

**Table 5:** Serologic response of hamsters following infection with *St. Louis encephalitis virus* (SLEV) strain Be Ar 23379 and subsequent challenge with *West Nile virus* (WNV)

Hamster no.	HI antibody 30 days after SLEV infection		HI antibody titer 6 days WNV challenge		WN MAC-ELISA 6 days after WNV challenge
	SLEV	WNV	SLEV	WNV	
8276	NT	NT	1:320	1:640	0.202^a^
8277	NT	NT	1:320	1:640	0.185
8278	1:80	1:80	1:160	1:160	0.141
8279	1:80	1:80	1:160	1:160	0.165
8280	1:80	1:80	1:640	1:640	0.276
8281	1:40	1:20	1:640	1:640	0.555
8282	1:80	1:40	1:160	1:80	0.177
8283	1:80	1:80	1:160	1:160	0.166
8298	1:160	1:80	1:320	1:320	0.139
8299	1:320	1:320	1:640	1:640	0.240

NT = not tested. HI = hemagglutination inhibition. ^a^Optical density value (>0.200 is positive).

Titration of daily blood samples from the SLEV-immune hamsters gave results similar to those in the JEV-immune animals. After challenge with WNV, 7 of the 10 SLEV-immune hamsters had brief, low-level viremia (Figure). However, three hamsters had no detectable viremia.

Serologic studies on blood samples taken 30 days after SLEV infection indicated that all the tested animals had been infected (Table 5). The HI response at 30 days was characteristic of primary *Flavivirus* infection. Six days after WNV infection, HI antibody titers had increased, indicating a secondary flavivirus antibody response. As with the JEV-immune hamsters, the IgM response of the SLEV-immune animals was minimal following the second flavivirus (WNV) infection (Tables 4,5).

Consistent with the low levels of WNV viremia (Figure), all the SLEV-immune hamsters (n = 32) survived subsequent challenge with WNV (Table 3). These animals did not appear clinically ill. These results indicate that prior immunity to SLEV also protected the hamsters from WNV encephalitis and death.

### Infection of YFV-Immune Hamsters with WNV

Based on the results obtained with JEV- and SLEV-immune hamsters, we tested the effect of prior immunization with a non-JEV serocomplex *Flavivirus* on subsequent WNV infection. Accordingly, a group of 30 hamsters was inoculated IP with 10^6.0^ TCID_50_ of the live attenuated 17D YFV strain. Thirty days after immunization, nine of the animals were bled and tested for HI antibodies to YFV and WNV (Table 6). Six days later (36 days after 17D vaccination), the hamsters were inoculated IP with 10^4^ TCID_50_ of WNV. Ten of these animals were bled daily for 6 consecutive days to determine the level of viremia and subsequent antibody response (Figure) (Table 6).

**Table 6:** Serologic response of hamsters following immunization with the 17D yellow fever (YF) vaccine and subsequent challenge with *West Nile virus* (WNV)

Hamster no.	HI antibody titer 30 days after YF immunization		HI antibody titer 6 days after WNV challenge		WN MAC-ELISA 6 days after WNV challenge
	YF	WNV	YF	WNV	
8226	1:20	1:20	1:320	1:320	0.783^a^
8227	1:40	1:20	1:320	1:320	0.484
8228	1:80	1:40	1:640	1:640	0.378
8229	<1:20	1:20	1:640	1:640	0.311
8230	<1:20	1:20	1:320	1:320	0.694
8231	1:40	1:20	1:640	1:640	0.511
8233	1:40	1:20	1:320	1:320	0.345
8234	1:20	1:20	1:640	1:640	0.418
8235	1:40	1:40	1:320	1:320	0.658

^a^Optical density value (>0.200 is positive). HI = hemagglutination inhibition.

Following challenge with WNV, YFV-immune hamsters had an intermediate level of viremia (Figure). The mean WNV titers in the YFV-immune hamsters were higher than in the JEV- and SLEV-immune groups, but the titers were lower than in the *Flavivirus*-naïve (control) hamsters. The death rate in the YFV-immune hamsters was also lower; 4 (13%) of 30 YFV-immune hamsters died after challenge with WNV, compared with 47% in the control group (Table 3).

The HI antibody response after vaccination with YFV 17-D virus (Table 6) was less intense than the primary antibody responses to the other three flaviviruses (Tables 1,2,4,5). Monath (*26*,*34*) also observed that immunization with 17-D virus induces a weaker HI and complement-fixing antibody response than infection with a wild-type YFV strain. Nonetheless, 6 days after challenge with WNV, the animals previously immunized with 17-D virus demonstrated a strong secondary-type *Flavivirus* antibody response. Interestingly, the 17-D immune animals also had a stronger IgM response to WNV infection. These data indicate that 17-D vaccine gives only partial protection against challenge with WNV.

## Discussion

The results of these hamster studies provide new information that may be useful in predicting the eventual geographic spread and public health importance of WNV in the Americas, as well as in developing novel methods for its control. The results also demonstrate the difficulty in making a serologic diagnosis of WNV infection in human or animal populations exposed to other flaviviruses.

First, our results clearly demonstrate that prior infection (and immunity) to JEV and SLEV protects hamsters from fatal WNV encephalitis (Table 3) and diminishes the severity of WNV infection (Figure). Other investigators (*17*–*20*) have reported similar findings in experimentally infected hamsters, pigs, and monkeys. The SA14-2-8 JEV strain used in our studies is one of several live attenuated JEV vaccines originally derived from the JEV SA14 wild-type parent strain (*35*,*36*); two of these vaccine derivatives, SA14-2-8 and SA14-14-2, have been widely used in China to immunize humans, equines, and pigs (*24*,*25*,*35*). Consequently, considerable information is already available on their biological and genetic characteristics, immunogenicity, safety, efficacy, and duration of immunity (*24*,*25*,*35*–*37*). The SA14 vaccine derivatives were obtained by serial passage (>100 times) in primary hamster kidney (PHK) cell cultures. Because the PHK cell substrate has not been approved by the World Health Organization as a vaccine substrate for use in humans, it is doubtful that the SA14 vaccine derivatives could be used in people in the United States or in other western countries. However, SA14-2-8 live attenuated JEV vaccine has been used successfully in >1 million horses in China (BQ Chen, pers. comm.) (*25*), and potentially it could be used in equines in the United States to protect against WNV encephalitis.

We are testing a commercial inactivated JEV vaccine (JE-VAX) that is already licensed for human use in the United States. If the licensed inactivated JEV vaccine protects hamsters in a manner similar to the SA14-2-8 attenuated vaccine, it might be considered as an interim WNV vaccine for groups of humans at high risk of exposure, such as laboratory workers and veterinarians, to protect against WNV encephalitis until a specific WNV vaccine is available. Several potential human WNV vaccines are now under development (*38*,*39*); however, it will probably be years before the testing and approval process is completed and they are licensed for human use.

A second potentially important finding from our hamster studies was that animals previously infected with JEV or SLEV viruses had a much lower viremia on challenge with WNV, compared with nonimmune animals (Table 1) (Figure). If a similar reduction in the level of viremia occurred in JEV- and SLEV-immune animals of other species (i.e., birds and pigs), such animals would probably be inefficient amplifying hosts for WNV virus. Interference from heterologous antibodies to other JEV-serocomplex viruses in birds and other vertebrate hosts may help explain the unique and largely nonoverlapping geographic distribution of the various members of this medically important *Flavivirus* complex (*40*,*41*). To date, the spread of WNV in North America has been limited to areas that are largely free of other endemic JEV complex flaviviruses (*41*–*43*). However, as WNV moves into South Florida and the Gulf Coast or into the Midwest, regions where SLEV is endemic (*43*), WNV could be restricted by heterologous antibodies to SLEV in the resident avian population. SLEV is also endemic in tropical America (*44*), so potentially the spread of WNV into that region might also be restricted for the same reason. It will be interesting to observe how this natural experiment unfolds.

A third important finding of our study concerns the difficulty in making a serologic diagnosis of recent WNV infection. The antigenic cross-reactivity of *Flavivirus* antibodies is well known, especially after a second or sequential *Flavivirus* infection in the same host (*11*–*15*). As noted, until now most WNV infections in humans and animals in North America have occurred in areas largely free of SLEV. In the northeastern region of the United States, serologic diagnosis of recent WNV infection has been relatively easy, since most people and animals were experiencing their first *Flavivirus* infection. However, as WNV spreads into geographic regions where people and animals have other preexisting *Flavivirus* antibodies (i.e., SLEV, YFV, DENV), the interpretation of HI, MAC-ELISA, and even neutralization test results will be more difficult. As we have shown (Tables 4, 5, and 6), hamsters with prior immunity to JEV, SLEV, or YFV had a broadly reacting HI antibody response after a second (sequential) WNV infection. Most of the JEV- and SLEV-immune hamsters did not develop specific IgM antibodies after WNV infection. Consequently, the WNV MAC-ELISA also may be of little diagnostic value in such human or animal cases. The HI test and MAC-ELISA are the two serologic tests most commonly used by public health and veterinary diagnostic laboratories in the United States to screen for WNV infection (*42*). Our data suggest that these tests may give equivocal results in regions where more than one *Flavivirus* is active and that other, more specific diagnostic techniques are needed.
